# Breakfast Skipping is associated with More Deleterious Lifestyle Behaviors among Japanese Men: The TRF-Japan Study Using the Original “Taberhythm” Smartphone Application

**DOI:** 10.1016/j.cdnut.2023.101977

**Published:** 2023-07-24

**Authors:** Koichiro Azuma, Motoko Kawashima, Tetsuya Nojiri, Kazuki Hamada, Masahiko Ayaki, Kazuo Tsubota, TRF-Japan Study Group, Kazuo Tsubota, Motoko Kawashima, Masahiko Ayaki, Koichiro Azuma, Tetsuya Nojiri, Akiyoshi Hanai, Kazuki Hamada, Shota Narisawa, Mitsuo Ishikawa, Daisuke Matsuoka

**Affiliations:** 7 Department of Ophthalmology, Keio University School of Medicine, Japan; 8 Tsubota Laboratory Incorporation, Tokyo, Japan; 9 Department of Ophthalmology, Keio University School of Medicine, Tokyo, Japan; 10 Institute for Integrated Sports Medicine, Keio University School of Medicine, Tokyo, Japan; 11 Oishi Kenko Incorporated, Tokyo, Japan; 1 Department of Medicine, Nerima General Hospital and Institute of Healthcare Quality Improvement, Public Interest Incorporated Foundation Tokyo Healthcare Foundation, Tokyo, Japan; 2 Sports Medicine Research Center, Keio University, Kanagawa, Japan; 3 Kawashima Ophthalmology Clinic, Saitama, Japan; 4 Department of Ophthalmology, Keio University School of Medicine, Tokyo, Japan; 5 Oishi Kenko Incorporated, Tokyo, Japan; 6 Tsubota Laboratory Incorporated, Tokyo, Japan

**Keywords:** breakfast skipping, Japanese, smartphone application, sex differences, obesity

## Abstract

**Background:**

Time-restricted eating has been increasingly recognized as a promising option to reduce food intake and combat obesity. Especially in Asian countries such as Japan, because of the wide variety of food choices available, a dietary approach that emphasizes meal timing can be more practical and easier to implement and adhere to, compared with approaches that focus on specific dietary content, such as low-fat or low-carbohydrate diets.

**Objectives:**

We aimed to identify eating patterns among Japanese men and women using a smartphone application (app) called “Taberhythm.” In addition, we sought to evaluate the relationship of breakfast eating habits with lifestyle behaviors and body mass index, and determine whether sex differences were present.

**Methods:**

A total of 3369 smartphone users were eligible to participate in this observational study. Users recorded 1 mo of lifestyle logs using the app; 254 participants (178 women, 38 ± 12 y old, body mass index 23.3 ± 4.9 kg/m^2^) had sufficient records to calculate daily fasting duration and sleep duration, and were eligible for the analyses.

**Results:**

Fasting duration was ∼12.6 h and was longer in women than men, among participants who never skipped breakfast. Breakfast skipping was associated with longer screen time, and more frequent snacking, only in men. Men with irregular breakfast eating patterns had a longer duration of fasting after awakening that was associated with obesity.

**Conclusions:**

We investigated eating patterns among Japanese people using a smartphone app and revealed that skipping breakfast was more deleterious in men than in women.

## Introduction

Time-restricted eating (TRE), which restricts the time of caloric intake to a window of 8–12 h without altering of the quantity or quality of the diet, can provide pleiotropic physiological benefits such as increased metabolic rate, reduced hepatic glucose production, and reduced macrophage infiltration in adipose tissue observed in rodents [1,2] and is becoming popular as an option to normalize energy balance [3,4]. Many individuals consume energy in greater amounts than expended, which is a global problem that has led to an obesity pandemic [5].

Until recently, methods to monitor human nutrition have been subjective, involving methods such as the 24-h food recall or FFQs. The increasing rate of smartphones use presents an opportunity to objectively monitor human nutrition and lifestyle [6]. The applicability of TRE in human health has been demonstrated [[7], [8], [9]]. Using their original smartphone application (app), Gill et al. [8] showed that human eating patterns could be measured and TRE could effectively reduce caloric intake, with subsequent weight loss [9].

Irregular eating patterns are associated with impaired cardiometabolic profiles, with skipping breakfast being one of the most prevalent irregular eating patterns [10,11]. Skipping breakfast has been linked to a higher prevalence of several cardiovascular and metabolic risk factors, such as overweight and central obesity [12,13]. It remains controversial whether skipping breakfast is a favorable option to maximize TRE [14,15].

Sex differences in response to fasting have been identified, with women tending to eat less after fasting and men eating more after fasting [16]. Levels of reproductive hormones, such as androgens and estrogens, are affected differently by intermittent fasting or the timing of caloric intake within a day. Fasting may decrease androgens whereas estrogen levels are not altered [17]. In women with polycystic ovary syndrome, earlier timing of caloric intake within the day contributes to a decrease in serum testosterone levels by 50%, compared with later timing of caloric intake [18]. Therefore, prolonged fasting after awakening or meal timing within the day may have sex-specific impacts.

The “Taberhythm” smartphone app was developed by Oishi Kenko Inc. [19]. The app was designed to investigate eating patterns, particularly the association between TRE and body weight and subjective feelings of happiness, and with data collection on dry eye and other conditions. In this study, we sought to identify eating patterns among Japanese individuals and fasting duration, and to evaluate the relationship of breakfast eating habits with lifestyle behaviors and BMI, with a particular focus on sex differences.

## Methods

### Participants

This observational study was performed as part of the TRF-Japan study, which aims to investigate healthier eating patterns among Japanese people, especially focusing on the effect of TRE on body weight and eye health [20,21]. Participants were included if they were iPhone users aged 20 y or older, and excluded if they were a nonresident in Japan. The recruitment channels for participants included the Apple App Store and Twitter, so the study mainly included individuals familiar with smartphones and their apps.

Women and men aged 20 y or older were recruited via a website. A total of 5756 smartphone users installed the Taberhythm app. Among them, 2554 participants had at least one record on the app between September 2018 and March 2020. Participants were asked to record all foods and calorie-containing beverages consumed, along with their wake-up times, bedtimes, subjective feelings of happiness, and eye symptoms.

**FIGURE 1:**
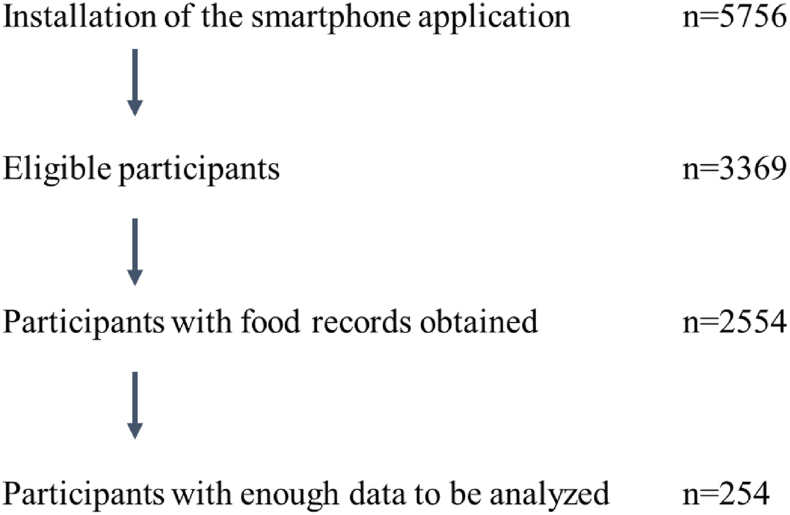
Description of participant recruitment. To calculate daily fating duration and sleep duration, records of dinner time, bedtime, subsequent wake-up time, and breakfast time are required and considered sufficient for analysis.

The data were mostly incomplete; to calculate daily fasting duration and sleep duration, data for dinner time, bedtime, subsequent waking time, and breakfast time were needed and considered sufficient for analysis. We also excluded pregnant women and shift workers, yielding a total of 254 participants included in the analysis (Figure 1).

Because this was a noninvasive, web-based observational study, informed consent was not obtained from study participants. Instead, on the first screen of the smartphone app, we included a statement that this was a research app and all data obtained would be used for the study. We also clearly stated that participation was completely voluntary, participants could withdraw at any time, and participants’ anonymity would be assured. Only after participants pressed the “Agree” button at the bottom of the screen could they proceed to the next screen of the app. We also provided contact information about the study and an inquiry form on the app. This opt-out study was approved by the institutional review board of Keio University School of Medicine (no. 20170162).

### Taberhythm smartphone application

The Taberhythm smartphone iOS app (Supplemental Figure 1), written in Japanese, was developed by Oishi Kenko Inc. and is available from the App Store (Apple Inc.) to adults aged 20 y or older living in Japan. Once participants had electronically agreed to participate in the study, they could fully access the app. All participant data were transferred to a web server and analyzed for this study [19]. Self-reported weight, height, and screen time were required inputs for study participation, and were used for analyses.

### Definition of mealtimes

Mealtimes were recorded separately by manually selecting a meal category from among 5 categories: breakfast, lunch, dinner, snack, and drink (except non-calorie-containing beverages). The times for the above meals were recorded when a photo of the meal was taken. There was also an option to record meals by manually selecting the time, in min, without a meal photo.

All data for each record were transferred to a web server, and feedback notifications were sent a maximum of 5 times a day to avoid forgetting to record meals, wake-up times, and bedtimes. Tapping each notification would open the app screen and prompt the user to record the respective time for wake-up, breakfast, lunch, dinner, and bedtime. The default notification timings were set as follows: 7:00 AM for recording the wake-up time and measuring body weight, 7:30 AM for breakfast, 12:30 PM for lunch, 8:00 PM for dinner, and 12:00 AM for reflecting on the day. Users had the flexibility to individually set and delete feedback timings.

### Definition of daily eating duration

Daily eating duration was defined as the duration, in h, between breakfast (or the first caloric intake after 5 AM and before breakfast) and the last caloric intake (including snacks) before 5 AM of the next day.

### Measuring steps per day

As an objective assessment of physical activity, we used steps per day, recorded with participants’ smartphones. The data were acquired using the iOS basic application Health Care. However, it was not mandatory for participants to grant us access to their step count data. As a result, the data were obtained from only a subset of participants. There was no option to record this measure manually.

### Statistical analyses

Because there were large differences in the amount of raw data for each participant, data for each participant are expressed as averaged data.

Because we wished to focus on sex differences and daily fasting duration, data were analyzed according to sex and breakfast eating habits because skipping breakfast has been associated with daily fasting duration.

The Shapiro-Wilk test was used to check the data normality. In cases where the data were not normally distributed, the Mann-Whitney U test was used instead of 1-factor ANOVA to assess group differences: men compared with women, and everyday breakfast consumers compared with breakfast skippers within each sex. Linear regression analyses were performed for variables related to clinical characteristics and eating patterns, excluding age and BMI. Age and BMI were adjusted for in the models of these analyses to assess group differences. Associations between variables were analyzed using the Spearman test with IBM SPSS Statistics version 21.0 (IBM Corp.). *P* values <0.05 were considered statistically significant.

## Results

### Clinical characteristics

As shown in Table 1, women were slightly younger (36 ± 12 compared with 42 ± 12 y old, *P <* 0.001) and leaner (22.6 ± 4.9 compared with 24.9 ± 4.6, *P <* 0.01) than men.

**TABLE 1:** Clinical characteristics of participants

	Women (*n* = 178)	Men (*n =* 76)
Age (y)	36 ± 12	42±12∗∗
BMI (kg/m^2^)	22.6 ± 4.9	24.9±4.6∗∗
Screen time (h)	6.6 ± 3.5	7.5 ± 3.7
Steps per day	5690 ± 3250 (*n* = 154)	7434 ± 3667∗∗ (*n* = 66)
Alcohol drink (%)	19%	29%
Sleep duration (min)	424 ± 81	402 ± 73
Breakfast time (h)	8.2 ± 1.2	8.1 ± 1.2
Lunch time (h)	12.8 ± 1.0	13.0 ± 0.9
Dinner time (h)	19.7 ± 1.4	20.2 ± 1.1∗
%Breakfast eating (%)	82 ± 30	82 ± 28
Snacks per day	0.65 ± 0.70	0.35 ± 0.46∗∗
Fasting duration (min)	772 ± 129	732 ± 121
Fasting duration before sleep (min)	247 ± 81	233 ± 83
Fasting duration after wake-up (min)	102 ± 85	97 ± 85

∗*P* < 0.05, ∗∗*P* < 0.01 vs. women using 1-factor ANOVA (age and BMI), and linear regression analyses adjusted for age and BMI in the models.

Breakfast time, lunch time, and dinner time were expressed as hours since midnight.

Alcohol consumption was less prevalent among women than men (19% compared with 29%, *P <* 0.05), and sleep duration and fasting duration were longer among women than men (424 ± 81 compared with 402 ± 72 min and 772 ± 129 compared with 732 ± 121 min, respectively; both *P <* 0.05). However, after adjusting for age and BMI, these sex differences disappeared. Even after adjusting for age and BMI, dinner time was earlier in women than in men (19.7 ± 1.4 compared with 20.2 ± 1.1, *P <* 0.05) and women ate snacks approximately twice as frequently as men (0.65 ± 0.70 compared with 0.35 ± 0.46 times/d, *P <* 0.01) and had fewer steps per day (5690 ± 3250 compared with 7434 ± 3667, *P <* 0.01).

### Mealtimes

**FIGURE 2:**
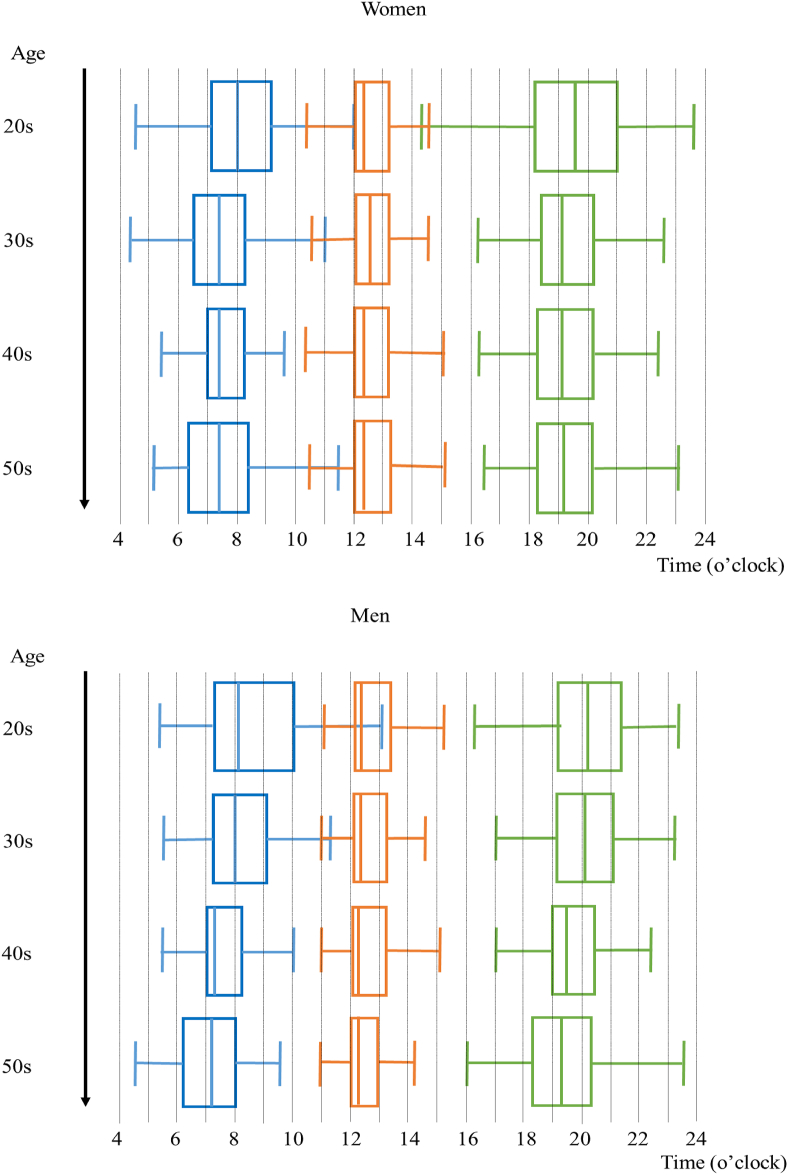
Dietary pattern by sex and age. Distributions of all logged data for breakfast time, lunch time, and dinner time are shown as box plots, by sex and age groups (breakfast: blue boxes, lunch: red boxes, dinner: green boxes).

All the raw data for mealtimes are shown as box plots by sex and age groups (Figure 2). In men, aging was associated with earlier breakfast times (ρ *=* −0.42, *P <* 0.001) but less so with the timing of subsequent meals (ρ *=* −0.30, *P <* 0.01 for lunch time and ρ *=* −0.25, *P <* 0.05 for dinner time). In women, the aging trends were less clear (ρ *=* −0.22, *P <* 0.01 for breakfast and ρ *=* −0.05, and −0.13; both nonsignificant (n.s.) for lunch and dinner, respectively).

**FIGURE 3:**
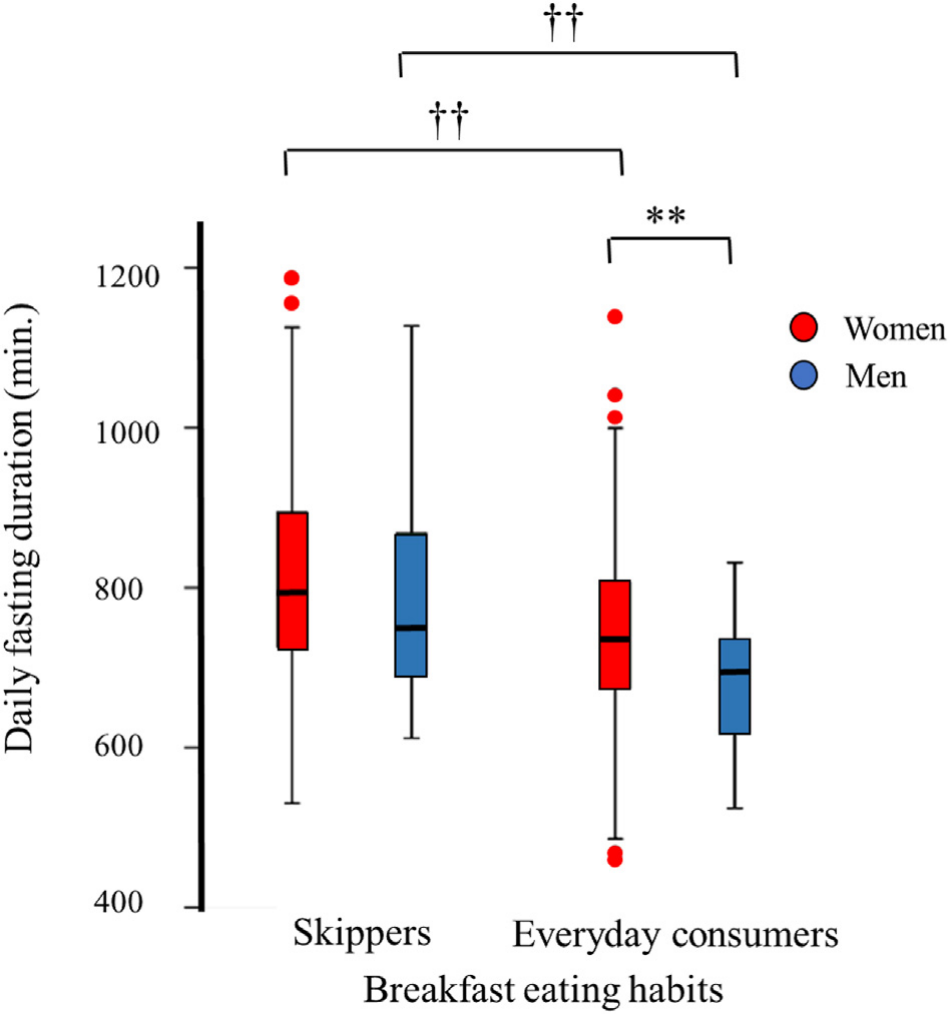
Daily fasting duration in relation to breakfast eating habits. Distributions of daily fasting duration are shown by sex (women: red columns, men: blue columns) and breakfast eating habits. ∗∗*P* < 0.01 vs. women, ^††^*P* < 0.01 vs. breakfast skippers, using linear regression analyses adjusted for age and BMI in the models.

As shown in Figure 3, breakfast skippers or irregular breakfast eaters (that is, percentage of breakfast eating <100%) had longer daily fasting duration than everyday breakfast consumers in both women and men (816 ± 133 compared with 742 ± 118 min, *P <* 0.01 for women, and 786 ± 136 compared with 682 ± 77 min, *P <* 0.01 for men). Therefore, we performed analyses by sex and breakfast eating habits (Table 2).

**TABLE 2:** Clinical characteristics by breakfast eating habits

	Breakfast skippers		Everyday breakfast consumers	
	Women (*n* = 71)	Men (*n* = 37)	Women (*n* = 107)	Men (*n* = 39)
Age (y)	35 ± 12	40 ± 12∗	36 ± 12	43 ± 12∗∗
BMI (kg/m^2^)	22.7 ± 5.3	25.3 ± 5.0∗	22.6 ± 4.6	24.5 ± 4.1∗
Screen time (h)	7.0 ± 3.3	8.9 ± 3.4∗∗	6.3 ± 3.6	6.3 ± 3.5^††^
Steps per day	5857 ± 2840 (*n* = 63)	7286 ± 3255∗∗ (*n* = 32)	5575 ± 3517 (*n* = 91)	7573 ± 4061∗∗ (*n* = 34)
Alcohol drink (%)	24%	34%	15%	26%
Sleep duration (min)	429 ± 87	390 ± 75	420 ± 77	415 ± 70
Breakfast time (h)	8.7 ± 1.1	8.5 ± 1.4	7.9 ± 1.2^††^	7.8±1.0^†^
Lunch time (h)	13.0 ± 0.9	13.0 ± 0.9	12.7 ± 1.0	12.9 ± 1.0
Dinner time (h)	20.1 ± 1.4	20.2 ± 1.1	19.4 ± 1.3^††^	20.1 ± 1.0∗∗
%Breakfast eating (%)	56%	63%		
Snacks per day	0.65 ± 0.64	0.46 ± 0.52	0.65 ± 0.73	0.25 ± 0.38∗∗^†^
Fasting duration (min)	816 ± 133	786 ± 136	742 ± 118^††^	682 ± 77^††^∗∗
Fasting duration before sleep (min)	251 ± 78	261 ± 83	244 ± 83	208 ± 76^††^∗
Fasting duration after wake-up (min)	136 ± 94	136 ± 103	79 ± 70^††^	60 ± 34^††^

∗*P* < 0.05, ∗∗*P* < 0.01 vs. women using 1-factor ANOVA (age and BMI), and linear regression analyses adjusted for age and BMI in the models.

^†^*P* < 0.05.

^††^*P* < 0.01 vs. breakfast skippers using 1-factor ANOVA (age and BMI), and linear regression analyses adjusted for age and BMI.

Breakfast time, lunch time, and dinner time were expressed as hours since midnight.

### Physical activity

Because there was a significant sex difference in daily step count, correlation analyses were performed separately for women and men. In both groups, no association was found between daily step count and daily fasting duration (ρ *=* −0.12, n.s. in men; ρ *=* 0.07, n.s. in women), screen time (ρ *=* 0.05, n.s. in men; ρ *=* 0.12, n.s. in women), breakfast eating habits (ρ *=* 0.03, n.s. in men; ρ *=* −0.08, n.s. in women), or BMI (ρ *=* −0.04, n.s. in men; ρ *=* −0.12, n.s. in women).

### Clinical characteristics by breakfast eating habits

**FIGURE 4:**
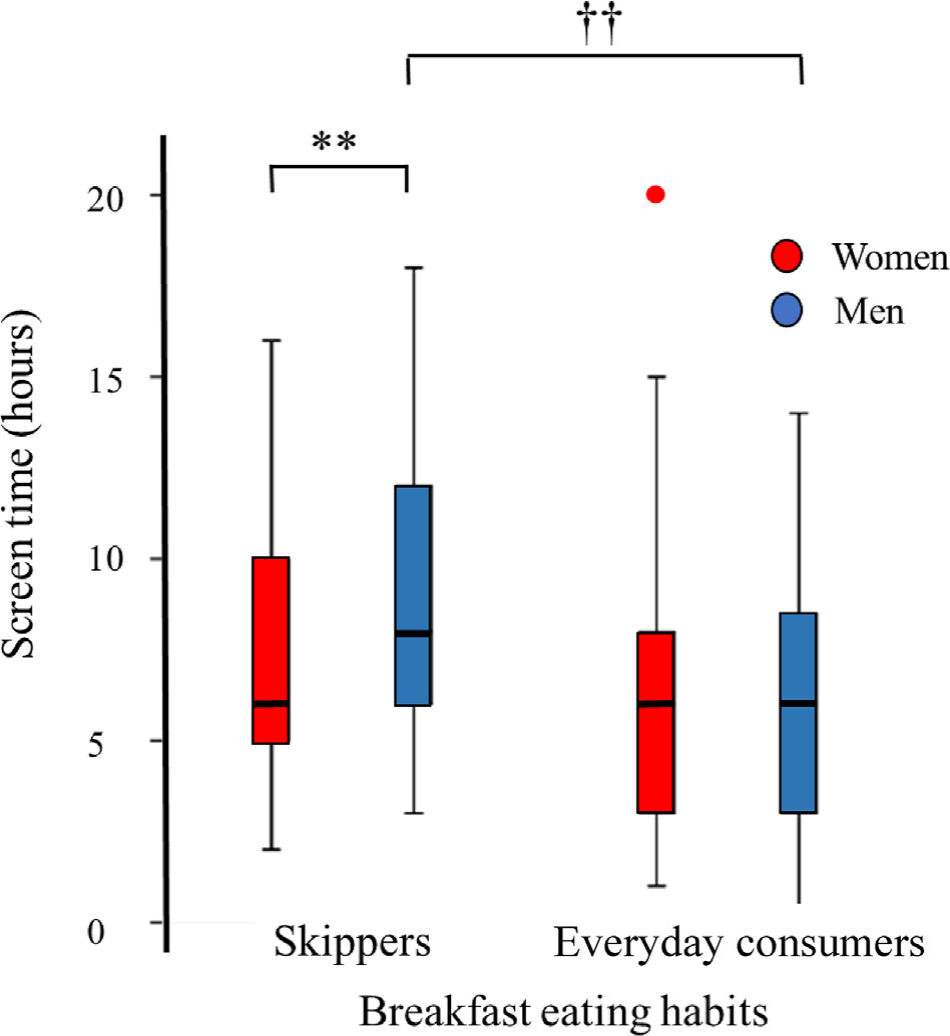
Screen time by sex and breakfast eating habits. Distributions of daily screen time (duration) are shown by sex (women: red columns, men: blue columns) and breakfast eating habits. ∗∗*P* < 0.01 vs. women, ^††^*P* < 0.01 vs. breakfast skippers, using linear regression analyses adjusted for age and BMI in the models.

There was no significant difference in sex, age, and BMI between breakfast skippers or irregular breakfast eaters (breakfast skippers) and everyday breakfast consumers. Screen time was significantly longer among men with breakfast skipping (*P <* 0.01), and there was a clear sex interaction of breakfast eating habits with screen time (Figure 4, *P <* 0.05). Among women, dinner time was earlier among everyday breakfast consumers than those who skipped breakfast, and there was no difference in dinner time among men according to breakfast eating habits. Subsequently, among everyday breakfast consumers, women had longer fasting duration before bedtime and longer daily fasting duration than men (both *P <* 0.01). Among men, the frequency of eating snacks was significantly lower among everyday breakfast consumers than among those who skipped breakfast (Table 2).

### Associations with fasting duration

As shown in Table 3, among everyday breakfast consumers, there was a clear negative association between dinner time and fasting duration (ρ *=* −0.45, *P <* 0.01 in men; ρ *=* −0.39, *P <* 0.01 in women), whereas the association was less clear in breakfast skippers (ρ *=* −0.21, n.s. in men; ρ *=* −0.32, *P <* 0.01 in women).

**TABLE 3:** Association of eating patterns with daily fasting duration

	Correlation coefficients			
	Breakfast skippers		Everyday breakfast consumers	
	Women	Men	Women	Men
Breakfast time	0.31∗	0.44∗	0.15	0.31
Lunch time	0.09	0.35∗	0.08	0.27
Dinner time	−0.32∗∗	−0.21	−0.39∗∗	−0.45∗∗
Snacks per day	−0.49∗∗	−0.09	−0.22∗	−0.18

∗*P* < 0.05, ∗∗*P* < 0.01, by Spearman test.

The frequency of eating snacks was negatively associated with fasting duration only in women, especially among breakfast skippers (ρ *=* −0.49, *P <* 0.01 in women skipping breakfast; ρ *=* −0.22, *P <* 0.05 in women consuming breakfast every day). There was no significant association in men (ρ *=* −0.09, n.s. in men skipping breakfast; ρ *=* −0.18, n.s., in men consuming breakfast every day).

### Associations with BMI

As shown in Table 4, among men skipping breakfast, there was a positive association with postawakening fasting duration with BMI (ρ = 0.39, *P <* 0.05); there was no association among men consuming breakfast every day (ρ *=* 0.17, n.s.). In women, there was a weak negative association among everyday breakfast consumers (ρ *=* −0.21, *P <* 0.05), and there was no association among breakfast skippers (ρ *=* −0.04, n.s.).

**TABLE 4:** Association of daily fasting duration with BMI

	Correlation coefficients			
	Breakfast skippers		Everyday breakfast consumers	
	Women	Men	Women	Men
Daily fasting duration	−0.05	0.08	−0.06	0.04
Fasting duration before sleep	−0.08	−0.13	−0.04	0.31
Fasting duration after wake-up	−0.04	0.39∗	−0.21∗	0.17

∗*P* < 0.05, by Spearman test.

## Discussion

In the present lifestyle log study using a smartphone app, the most striking sex differences in dietary behaviors were the deleterious effects of skipping breakfast. Longer screen time, and more frequent snacking were associated with breakfast skipping only in men. In women, dinner time was later among breakfast skippers, although there was an unclear deleterious effect of longer fasting time after waking up, such as an association with higher BMI.

Using an original smartphone app, Gill et al. [8] found that daily fasting duration was <10 h (600 min) in most healthy adults in the United States. Moreover, when overweight individuals with <10 h fasting duration were advised to eat for only 10–11 h (fasting duration of 13–14 h) daily for 16 wk, they successfully reduced body weight [8]. Most of our participants ate for a much shorter duration and their fasting duration was longer than 10 h; this was much longer among breakfast skippers. Among everyday breakfast consumers, women ate for a shorter duration (longer fasting duration) than men. Because participants in this study were much leaner than those in Gill et al.’s [8] study and women were leaner than in men in this study, the longer fasting duration in our study may indicate its potential metabolic effect on body weight. However, we did not observe any significant association between fasting duration and body weight in our study. This lack of association could possibly be attributed to the relatively homogenous and narrow range of BMI within our study population. Moreover, it is likely that differences in race and ethnicity also influenced the results.

Sakurai et al. [22] reported that breakfast skippers gained more weight or had larger increases in waist circumference than everyday breakfast consumers in men, but not in women. We also found that skipping breakfast was associated with longer screen time only in men, and fasting duration after waking up was associated with higher BMI in men. One explanation is a sex difference for obesity; the mean body weight of young and middle-aged women has decreased during these 40 y in Japan, according to the National Health and Nutrition Survey. Therefore, we conducted subanalyzes, excluding women with BMI <22 kg/m^2^; however, the results remained unchanged (Supplemental Table 1).

Zandian et al. [16] assessed the response to fasting among lean young women and men to determine whether sex differences were present. They reported that women consumed 12% less food after fasting, whereas men ate 28% more food after fasting. Women who ate at a nearly constant rate (linear eaters) consumed less food than those who ate at an initially high speed that decreased over the course of a meal (decelerated eaters) [16]. Women decreased their food intake after fasting because their eating pattern became more linear, whereas men increased their food intake after fasting because the rate at which they ate became more decelerated [16]. This is a possible plausible reason for the lower deleterious effect of breakfast skipping in women. A literature review conducted by Cienfuegos et al. [17] reported that fasting may decrease androgens whereas estrogen levels are not altered. This decrease in androgens in men could have a negative impact on metabolic health, whereas in women, the effect is the opposite [23]. In fact, a study involving women with polycystic ovary syndrome who practiced TRE from 1:00 PM to 9:00 PM (8 h) for 6 wk demonstrated a decrease in androgen levels, significant weight loss, and improved insulin sensitivity [24].

We must acknowledge the limitation of selection bias in our study; only 5% (254 of 5756) of potential participants were eligible because they provided enough data to be analyzed. However, this reflects a more real life situation than a web-based survey and the data reflect participants’ initiative, as compared with data obtained in a research setting with compensation and high adherence. Because of our small sample size and incomplete or missing data, conducting more detailed analyses was difficult. Guinter et al. [25] reported that women who never ate breakfast as well as women with regular breakfast consumption were less likely to be obese or to gain weight as compared with women who ate breakfast 3–4 d per wk [25]. Therefore, breakfast skippers should ideally be divided into never breakfast eaters and irregular breakfast eaters. However, owing to our small sample size, we only divided participants into everyday breakfast consumers and breakfast skippers, with the latter including never breakfast eaters. In recruiting subjects, we employed an open participation approach rather than implementing equal allocation based on sex. The recruitment channels utilized were the Apple App Store and Twitter with the assumption that any sex bias resulting from these channels would be minimal. Therefore, the disparity in the number of participants by sex can be primarily attributed to a higher level of interest among women in this app pertaining to lifestyle rhythms and weight management. Regarding the potential impact on the results, it is worth noting that women’s higher participation could be attributed to their generally higher level of health consciousness compared with men. This difference in health consciousness may have influence the manifestation of lifestyle habits among women mentioned in this study, potentially leading to a lack of significant findings when compared with men. Also, as participants were not required to grant us access to their step count data, the data were obtained from only a subset of participants. Furthermore, because of the fact that not all participants, particularly women, wore their smartphone at all times, the number of steps per day was not accurate, which is the reason for finding no association of steps per day with positive behaviors. Although smartphone step counting can serve as a proxy of physical activity, it has limitations. Step count alone cannot accurately assess activity intensity, making it challenging to estimate the dose or energy expenditure associated with physical activity, which is crucial for understanding its health benefits [26]. Smartphone step counting primarily relies on sensors such as accelerometers and gyroscopes to detect walking movements. Other forms of activity such as running or cycling cannot be accurately measured. Finally, we could not determine the causal relationship of breakfast skipping with longer screen time, and possible higher BMI in men. A scientific statement from the AHA reviewed the cardiometabolic health effects of skipping breakfast [11]. The findings from interventions do not consistently demonstrate a positive effect of breakfast consumption on cardiometabolic risk. Instead, the association between skipping breakfast and unfavorable cardiometabolic health can be partially explained by the presence of other unhealthy lifestyle habits, such as late dinners, higher alcohol consumption, and infrequent exercise [11]. Furthermore, a cluster analysis conducted on representative samples of the Austrian population revealed that obesity, insomnia, depression, and self-rated poor health-status were more prevalent among a cluster of participants who reported longer fasting intervals and a high proportion of breakfast skippers [27]. Nevertheless, this study highlights the importance of further research to clarify whether breakfast skipping or delaying breakfast can mitigate the metabolic health benefits of TRF [15], as well as the contribution of screen time to eating habits and BMI, because updated evidence is still lacking for these emerging health issues in the digital era [28].

In conclusion, in the present lifestyle log study using a smartphone app, for the first time, we reported the eating pattern among Japanese people and identified a fasting duration of ∼12.6 h. Breakfast skipping was more deleterious among men and was associated with longer screen time, and more frequent snacking. The resultant longer fasting duration after awakening was associated with obesity among men with irregular patterns of eating breakfast.

## Funding

Oishi Kenko Incorporated developed the smartphone app and covered all expenses of developing the app, as well as the costs of processing and analyzing the data. The CEO and an employee of Oishi Kenko Incorporated had central roles in the collection and analysis of data. TN has received directors’ compensation from this company. KH received a salary as a full-time employee of Oishi Kenko Incorporated.

## Author contributions

The authors’ responsibilities were as follows—KT, TN, KA, MK, MA: designed the research; TN, MK, KH: conducted the research; TN, KH: provided essential materials; KA, MK, KH: analyzed data; KA, MK: wrote the paper; MA, KTL had primary responsibility for the final content; and all authors: read and approved the final manuscript.

## Conflict of Interest

This study was designed purely for academic interest and is completely unrelated to any employment, consultancy, patents, products in development, or marketed products of the company (Oishi Kenko Incorporated). The app was developed as a research tool; in the future, the app will be used to support users in maintaining healthier daily eating rhythms. This does not alter our adherence to Current Developments in Nutrition policies on sharing data and materials.

## Data availability

The data described in the manuscript, code book, and analytic code will be made available upon request, pending adequate permissions.

## Appendix A Supplementary data

Multimedia component1 Multimedia component1
