# Supplementary material for: Breakfast Skipping is associated with More Deleterious Lifestyle Behaviors among Japanese Men: The TRF-Japan Study Using the Original “Taberhythm” Smartphone Application
Source: Curr Dev Nutr. 2023 Jul 24;7(9):101977. doi: 10.1016/j.cdnut.2023.101977 (PMC10448026; doi:10.1016/j.cdnut.2023.101977)
Supplement: Multimedia component1 [file mmc1.docx]

**Supplemental Table 1 Clinical characteristics by sex and breakfast eating habits, excluding women with BMI <22 kg/m^2^**

|  | Breakfast skippers | | Everyday breakfast consumers | |
| --- | --- | --- | --- | --- |
|  | Women n=33 | Men  n=37 | Women n=48 | Men  n=39 |
| Age (years old) | 37±12 | 40±12 | 36±12 | 43±12** |
| BMI (kg/m^2^) | 26.4±5.8 | 25.3±5.0 | 26.3±4.7 | 24.5±4.1 |
| Screen time (hr) | 6.4±3.3 | 8.9±3.4** | 6.2±3.9 | 6.3±3.5^††^ |
| Steps per day | 4806±2201 n=28 | 7286±3255** n=32 | 5704±3879 n=40 | 7573±4061* n=34 |
| Alcohol drink (%) | 21% | 34% | 17% | 26% |
| Sleep duration (min) | 436±101 | 390±75* | 422±83 | 415±70 |
| Breakfast time (hr) | 8.6±1.0 | 8.5±1.4 | 7.7±1.2^††^ | 7.8±1.0^†^ |
| Lunch time (hr) | 12.9±0.8 | 13.0±0.9 | 12.8±1.2 | 12.9±1.0 |
| Dinner time (hr) | 19.9±1.1 | 20.2±1.1 | 19.5±1.5 | 20.1±1.0* |
| %Breakfast (%) | 60% | 63% |  |  |
| Snacks per day | 0.67±0.62 | 0.46±0.52 | 0.61±0.68 | 0.25±0.38**^†^ |
| Fasting duration (min) | 817±149 | 786±136 | 735±122^††^ | 682±77^††*^ |
| Fasting duration before sleep (min) | 246±71 | 261±83 | 246±86 | 208±76^††*^ |
| Fasting duration after wake-up (min) | 135±102 | 136±103 | 67±55^††^ | 60±34^††^ |

*p<0.05, **p<0.01 versus women using one-way analyses of variance (age and BMI), and linear regression analyses adjusted for age and BMI in the models.

^†^p<0.05, ^††^p<0.01 versus irregular breakfast eaters using one-way analyses of variance (age and BMI), and linear regression analyses adjusted for age and BMI.

Breakfast time, lunch time, and dinner time were expressed as hours since midnight.

BMI, body mass index.

**Supplemental Figure 1 Display of the Taberhythm smartphone iOS app**


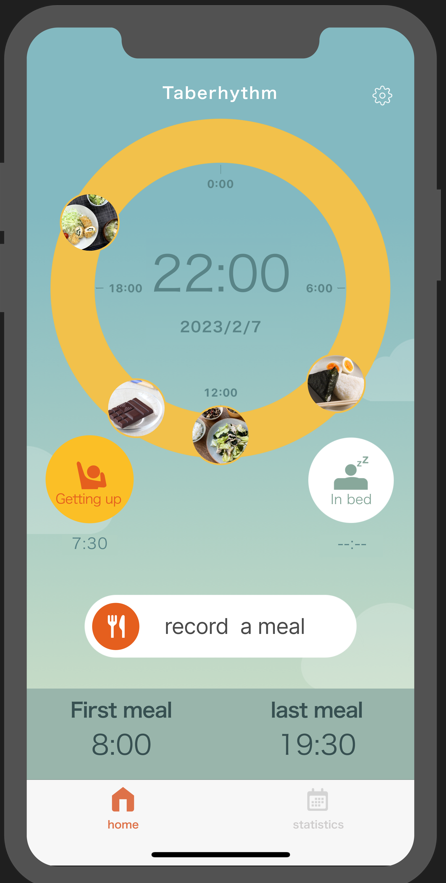


The letters were translated into English.
